# Functional Heterogeneity of Immune Cell Subpopulations in Atherosclerosis: From Basic Mechanisms to Precision Therapy

**DOI:** 10.31083/RCM49094

**Published:** 2026-08-21

**Authors:** Ming Li, Ce Bian, Bo Li

**Affiliations:** ^1^Department of Cardiology, Zibo Central Hospital Affiliated to Shandong Medical and Pharmaceutical University, 255036 Zibo, Shandong, China; ^2^Department of Cardiology, Zibo Central Hospital, 255036 Zibo, Shandong, China

**Keywords:** atherosclerosis, immune cell heterogeneity, macrophage polarization, T cell subsets, B cell targeting, precision therapy, immune microenvironment, foam cell, immune-metabolic crosstalk, plaque stability

## Abstract

Atherosclerotic cardiovascular disease (ASCVD) remains a leading cause of death worldwide. Accumulating evidence has established ASCVD as a lipid-driven chronic inflammatory disease in which the functional heterogeneity of immune cell subsets plays a pivotal role in plaque development and stability. This review systematically summarizes the dynamic immune microenvironment in atherosclerosis, moving beyond static cell categorization to explicitly link immune heterogeneity to the major stages of disease progression: lesion initiation, necrotic core expansion, fibrous cap weakening, and thrombotic complications. We further describe the continuous phenotypic shifts of key immune populations, particularly the transition of macrophages into specialized lipid-handling states and the context-dependent roles of distinct T-cell, B-cell, and innate immune cell subsets. By integrating recent advances in single-cell transcriptomics, we highlight how local molecular networks, cellular crosstalk, and impaired clearance of apoptotic cells govern plaque vulnerability. Furthermore, we critically evaluate spatiotemporal variations across vascular territories and the complex clinical implications of immune checkpoint regulation. Finally, this review discusses current barriers to clinical translation and explores the therapeutic potential of precision immunomodulatory strategies, aiming to bridge the gap between basic immunological mechanisms and targeted cardiovascular therapies.

## 1. Introduction

Atherosclerotic cardiovascular disease (ASCVD), primarily manifesting as ischemic heart disease and stroke, remains a leading cause of mortality and presents a substantial global health burden [1]. Despite optimal standard-of-care treatments, patients continue to face major clinical challenges, including residual cardiovascular risk, treatment resistance, and high rates of disease recurrence. These challenges underscore the limitations of focusing solely on traditional lipid-lowering therapies and highlight the urgent need to address the underlying pathobiology of the vascular wall.

Accumulating evidence has definitively established atherosclerosis as a lipid-driven chronic inflammatory disease. Recent technological advancements, particularly in single-cell transcriptomics and spatial profiling, have shifted the research paradigm by revealing the profound functional heterogeneity of immune cell subpopulations within the plaque microenvironment. The dynamic infiltration and phenotypic plasticity of these innate and adaptive immune cells serve as the core biological drivers of chronic vascular inflammation [2].

Therefore, elucidating this immune cell heterogeneity is essential for bridging basic mechanistic insights with clinical translation [1]. The objective of this review is to systematically evaluate the evolving characteristics of the immune microenvironment and explicitly map the functional plasticity of specific immune subsets to the major stages of atherosclerotic disease—from lesion initiation to plaque destabilization. By integrating these complex immune networks into a coherent pathobiologic framework, we aim to highlight the therapeutic potential of precision immunomodulatory strategies in stabilizing vulnerable plaques and mitigating ASCVD complications.

## 2. Atherosclerotic Immune Microenvironment: Inflammation, Infiltration, and Immunometabolism

### 2.1 Immunopathologic Basis of Vascular Inflammation

Atherosclerosis is recognized as a lipid-driven chronic inflammatory response characterized by the accumulation of lipids and immune cells within the arterial wall [3]. As the understanding of the disease has evolved from a “lipid-deposition” model to an “immune-microenvironment” framework, the functional heterogeneity of immune subsets has emerged as the primary driver of disease progression [4]. Within the plaque, specialized subsets, such as secreted phosphoprotein 1 (SPP1)^+^ macrophages, activate the nuclear factor kappa B (NF-κB) pathway via osteopontin (OPN) signaling, secreting pro-inflammatory cytokines including interleukin-6 (IL-6), tumor necrosis factor alpha (TNF-α), and C-C motif chemokine ligand 2 (CCL2) [5]. These factors induce vascular smooth muscle cells (VSMCs) to transition from a contractile to a synthetic phenotype, promoting pathological proliferation and matrix remodeling. This paracrine crosstalk creates a self-amplifying inflammatory loop between immune cells and the vascular wall cells.

### 2.2 Dynamics of Immune Infiltration Across Disease Stages

High-resolution single-cell analysis reveals that immune infiltration is a highly dynamic process, with the composition and functional state of immune cells shifting as lesions progress.

● Lesion Initiation: Early stages are dominated by the recruitment of classical monocytes and their differentiation into macrophages [6].

● Progression and Necrotic Core Expansion: As lipids accumulate, macrophages transition into specialized lipid-associated macrophage (LAM) states. The expansion of the necrotic core is driven by defective efferocytosis, while the accumulation of cholesterol crystals and cell-free DNA activates the NLR family pyrin domain-containing 3 (NLRP3) inflammasome and cyclic GMP-AMP synthase–stimulator of interferon genes (cGAS–STING) pathways [7,8].

● Plaque Destabilization: Vulnerable plaques are characterized by an influx of cytotoxic CD8^+^ T cells (notably Granzyme-K^+^ and Perforin^+^ populations) and neutrophils. These cells promote fibrous cap weakening by inducing VSMC apoptosis and secreting matrix metalloproteinases (MMPs) [9,10].

● Thrombotic Complications: Rupture or erosion triggers immunothrombosis, where Neutrophil Extracellular Traps (NETs) and platelets form a scaffold for occlusive thrombus formation [11].

Notably, current evidence suggests that the traditional M1/M2 polarization model is insufficient to describe plaque biology. Instead, macrophages exist along a continuous phenotypic spectrum dictated by local metabolic and inflammatory cues [6,12].

### 2.3 Immuno-Metabolic Crosstalk and Microenvironment Remodeling

The remodeling of the plaque microenvironment is inextricably linked to immune-metabolic interactions. Macrophages and T cells undergo metabolic reprogramming—favoring glycolysis in pro-inflammatory states and fatty acid oxidation in anti-inflammatory states—to adapt to the hypoxic and lipid-rich environment. Metabolic enzymes and intermediates function as critical signaling molecules that direct immune cell fate [13]. For instance, nutrient availability and lipid accumulation trigger metabolic switches that amplify inflammatory signaling [14]. Consequently, targeting these metabolic checkpoints offers a promising avenue for stabilizing the immune microenvironment and preventing plaque rupture [15].

## 3. Macrophage Heterogeneity: Orchestrating Lesion Evolution

Macrophages are the primary immune constituents of atherosclerotic plaques, driving disease progression through lipid processing, inflammatory signaling, and matrix remodeling. Rather than existing in discrete binary states, contemporary high-resolution evidence suggests that macrophages occupy a multidimensional functional continuum that evolves alongside the plaque microenvironment [16,17].

### 3.1 Beyond the M1/M2 Paradigm: The Functional Spectrum

While the traditional M1/M2 dichotomy provided an early foundational framework, single-cell RNA sequencing (scRNA-seq) has revealed a more complex landscape [17,18]. Macrophage phenotypes exist as a fluid continuum, including specialized populations such as LAMs and triggering receptor expressed on myeloid cells 2 (TREM2^+^) subsets adapted to handle lipid overload [19]. The disease trajectory reflects a transition from early-stage homeostatic programs to advanced, maladaptive inflammatory states. Understanding this plasticity is essential for developing precision therapies that target specific dysfunctional states rather than broad immune suppression [20].

### 3.2 Monocyte Recruitment and VSMC Transdifferentiation

Lesion initiation is driven by the recruitment of circulating monocytes, which differentiate into macrophages in response to local cues such as oxidized low-density lipoprotein (oxLDL) and chemokines [21]. Notably, the macrophage pool is not derived solely from hematopoietic sources; VSMCs exhibit significant plasticity, transdifferentiating into “macrophage-like” cells that contribute to the foam cell population and accelerate plaque growth [22,23,24]. This tissue-specific differentiation is governed by local hypoxia, lipid metabolites, and cellular crosstalk, resulting in at least four distinct functional clusters within advanced plaques [23,25].

### 3.3 The Multi-Layered Regulation of Foam Cell Formation

The transformation of macrophages into foam cells—the hallmark of atherosclerotic progression—is regulated by a synergistic network of lipid trafficking and metabolic reprogramming [26]:

● Lipid Influx and Cytoskeletal Dynamics: oxLDL internalization is mediated by scavenger receptors (CD36, scavenger receptor class A (SR-A)). Recent evidence highlights the role of intracellular osteopontin (iOPN) in inducing F-actin polymerization, which facilitates the membrane trafficking of CD36/SR-A and enhances lipid uptake [5].

● Cholesterol Efflux and Reverse Transport: The ATP-binding cassette transporter A1/G1 (ABCA1/ABCG1) pathway, regulated by the peroxisome proliferator-activated receptor gamma/signal transducer and activator of transcription 6 (PPARγ/STAT6) axis, serves as the primary antagonistic mechanism against lipid accumulation [27].

● Metabolic and Epigenetic Control: Metabolic shifts, such as protein phosphatase 1 regulatory subunit 3B (PPP1R3B)-mediated glycogenolysis, provide the energetic support required for cholesterol efflux [27,28]. Concurrently, epigenetic modifications, specifically N^6^-methyladenosine (m^6^A) methylation (e.g., via methyltransferase-like 3 (METTL3) or fat mass and obesity-associated protein (FTO)), modulate the stability of transcripts related to polarization and lipid metabolism, directly influencing foam cell formation efficiency [25].

### 3.4 Efferocytosis, Necrotic Core Expansion, and Plaque Stability

The transition from stable to vulnerable plaques is often marked by defective efferocytosis—the impaired clearance of apoptotic cells. In the advanced stages, pro-inflammatory signaling and oxidative stress downregulate phagocytic receptors like Mer receptor tyrosine kinase (MerTK), leading to secondary necrosis and the expansion of the necrotic core [29].

Furthermore, macrophage-derived signals directly influence the structural integrity of the fibrous cap. High-risk SPP1hi macrophages activate the NF-κB pathway and secrete matrix metalloproteinases (e.g., MMP9), which degrade the extracellular matrix [27]. Simultaneously, these cells produce cytokines that induce VSMCs to switch from a contractile to a synthetic phenotype, leading to a thinner, more rupture-prone fibrous cap [30]. Enhancing efferocytosis and stabilizing macrophage-VSMC crosstalk through pathways like PPARγ activation represent critical strategies for maintaining plaque stability [28].

## 4. T Cell Landscapes: Functional Plasticity and Clinical Paradoxes

T cells are central coordinators of the adaptive immune response in atherosclerosis. Their role evolves from initial recruitment during fatty streak formation to the direct induction of plaque rupture in advanced stages through cytotoxic activity and cytokine-mediated destabilization [31,32].

### 4.1 The Th1-Treg Axis: Orchestrating Chronic Inflammation

The progression of atherosclerosis is characterized by a shift in the CD4^+^ T cell compartment toward a pro-inflammatory Th1 dominance [33]. Driven by IL-12 and interferon gamma (IFN-γ), T-bet^+^ Th1 cells exacerbate local inflammation and promote macrophage activation [34]. Conversely, Foxp3^+^ regulatory T cells (Tregs) maintain vascular immune tolerance; however, their homeostatic role is often compromised in advanced lesions [35,36]. Clinical and preclinical data suggest that the Th17/Treg imbalance—rather than the presence of a single subset—is positively correlated with plaque severity. This suggests that the transition from stable to vulnerable lesions is dictated by the failure of local immunosuppressive programs to contain the Th1/Th17-driven response [37].

### 4.2 Cytotoxic CD8^+^ T Cells and Fibrous Cap Destabilization

CD8^+^ T cells exhibit significant clonal expansion within atherosclerotic plaques, particularly in advanced stages. High-resolution scRNA-seq profiling has identified a distinct population of Granzyme-K^+^ (GZMK^+^) and Perforin^+^ effector memory CD8^+^ T cells that infiltrate the fibrous cap [38]. These cells promote plaque instability through two primary mechanisms:

● Direct Cytotoxicity: Inducing apoptosis in smooth muscle cells (VSMCs) and macrophages via perforin/granzyme release [39,40].

● Paracrine Amplification: Secretion of IFN-γ, which suppresses collagen synthesis by VSMCs and polarizes macrophages toward a maladaptive, pro-inflammatory state. This cytotoxic infiltration is notably observed in aged murine models and high-risk human plaques, marking the transition from necrotic core expansion to imminent rupture [37,40,41].

### 4.3 Innate-Like T Cells: γδ T and NKT Cells

Non-classical T cells, including γδ T and Natural Killer T (NKT) cells, act as early sensors of the plaque microenvironment [42]. NKT cells recognize lipid antigens presented by CD1d and can rapidly modulate the Th1/Th2 balance [43]. While scRNA-seq confirms their presence in early fatty streaks, their role appears highly tissue-specific and dependent on the local cytokine and lipid milieu. Their rapid response suggests they serve as an “immunological bridge” between innate lipid sensing and the recruitment of the classical adaptive system [44].

### 4.4 The “Athero-Oncology” Paradox: Immune Checkpoints

The translation of immune checkpoint research from oncology to cardiovascular medicine has revealed a profound biological trade-off. While the programmed cell death protein 1/programmed death-ligand 1 (PD-1/PD-L1) and T-cell immunoreceptor with immunoglobulin and ITIM domains (TIGIT) pathways are essential for preventing T-cell overactivation in the vascular wall, their inhibition leads to rapid plaque progression. Clinical evidence from a retrospective matched-cohort study indicates that patients receiving immune checkpoint inhibitors (ICIs) have an approximately threefold higher risk of atherosclerotic cardiovascular events, including myocardial infarction, coronary revascularization, and ischemic stroke. In an imaging substudy, ICI therapy was also associated with accelerated progression of atherosclerotic plaque [45]. This “Athero-Oncology” paradox underscores that vascular immune tolerance is actively maintained by checkpoint signaling; therefore, any future immunomodulatory strategy must carefully balance systemic antitumor efficacy against the risk of acute vascular destabilization [46,47].

## 5. B Cell Subsets: Bidirectional Regulators of Vascular Homeostasis

B cells orchestrate the atherosclerotic landscape through a balance of protective and pathogenic signals. Their influence is mediated by subset-specific antibody production and sophisticated crosstalk with the T-cell compartment, evolving in complexity as the disease progresses from early lipid deposition to acute thrombotic events [48].

### 5.1 The B1-B2 Dichotomy: Protective vs. Pro-Atherogenic Signals

The functional heterogeneity of B cells is fundamentally tied to their ontogeny and specific antibody profiles.

Protective B1 and Marginal Zone B (MZB) Cells: Predominantly appearing in the early stages of lesion initiation, B1 cells secrete natural IgM antibodies that recognize oxidation-specific epitopes (OSEs) on oxLDL [49]. This neutralizing effect inhibits lipid uptake by macrophages and attenuates early inflammatory signaling. Similarly, MZB cells provide a protective layer, with their abundance inversely correlated with coronary artery disease severity in clinical cohorts [50].

Pro-atherogenic B2 and CD11c^+^ Subsets: In contrast, follicular B2 cells drive plaque progression by undergoing class-switch recombination to produce immunoglobulin G (IgG) and IgE [51]. These antibodies facilitate the activation of the classical complement pathway and exacerbate vascular endothelial damage. Notably, age-associated CD11c^+^ B cells significantly expand in advanced atherosclerosis, a process tightly linked to increased plaque burden and systemic inflammation [52].

### 5.2 Regulatory B Cells (Bregs) and Immune Tolerance

Bregs function as essential checkpoints to prevent excessive vascular inflammation. A distinct human memory B-cell subset (CD19^+^CD24^+^CD27^+^) has been identified to express high levels of TIGIT, which directly suppresses T-cell overactivation [53]. Bregs maintain immune homeostasis by secreting IL-10 and IL-4, the latter of which promotes a transition toward pro-resolving macrophage phenotypes [54]. In advanced metabolic stress, the follicular regulatory T (TFR) cell-Breg regulatory axis (comprising follicular regulatory T cells and Bregs) acts as a critical buffer, attempting to stabilize the immune microenvironment even as lipid-driven triggers intensify [55,56].

### 5.3 Pathological Antibody Dynamics and Immunothrombosis

B-cell-derived antibodies exhibit distinct spatiotemporal dynamics that influence plaque stability. While B1-derived IgM provides systemic protection, splenic B2-derived IgG antibodies have been shown to accumulate abnormally within plaques following myocardial infarction (MI) [51]. This accumulation accelerates post-ischemic atherosclerosis progression—a process confirmed in activation-induced cytidine deaminase (AID)-deficient mice, where the absence of IgG class-switching limits plaque expansion despite persistent hypercholesterolemia [57]. Beyond inflammation, these antibodies can mediate complement-dependent cytotoxicity, further weakening the structural integrity of the vascular wall and predisposing the plaque to rupture [58].

### 5.4 B-T Cell Crosstalk and Germinal Center Responses

The progression of chronic vascular inflammation is sustained by a functional interactive network between B and T cells [59].

● Antigen Presentation: B cells act as professional antigen-presenting cells (APCs), utilizing their B-cell receptors (BCR) to internalize OSEs and activate T cells through major histocompatibility complex class II (MHC-II) presentation [57].

● The Tfh-B2 Axis: In the germinal centers of the spleen and perivascular tertiary lymphoid structures, the interaction between Follicular Helper T (Tfh) cells and B2 cells drives the production of high-affinity autoantibodies [60,61].

● Innate-Adaptive Integration: B-cell activation via Toll-like receptors (TLRs)—specifically through the myeloid differentiation primary response 88 (MyD88) signaling pathway—synergistically amplifies T-cell recruitment. Conversely, specialized recruitment via the C-C chemokine receptor type 6 (CCR6) receptor on B1 cells ensures a steady supply of natural IgM to the vascular intima, providing a continuous feedback loop that modulates the intensity of the adaptive T-cell response [40,61,62].

## 6. Emerging Regulators: Bridging Innate Sensing and Plaque Rupture

Beyond the classical macrophage and lymphocyte axes, specialized innate populations—including dendritic cells, neutrophils, mast cells, and innate lymphoid cells—function as critical sensors and effectors that dictate the tempo of vascular inflammation [63,64,65].

### 6.1 Dendritic Cells: The Initiation Bridge

Dendritic cells (DCs) serve as the primary hub connecting innate lipid sensing to adaptive T-cell responses. During lesion initiation, DCs recognize atherosclerosis-related autoantigens (such as aldehyde dehydrogenase 4 family member A1 (ALDH4A1)) and regulate the balance between immune tolerance and activation. While tolerogenic DCs (TolDCs) maintain homeostasis by inducing Treg expansion and secreting IL-10, the hypercholesterolemic microenvironment drives DC maturation [32]. Mature DCs highly express costimulatory molecules (CD80/CD86) and secrete IL-6 and IFN-γ, effectively “priming” the vascular wall for chronic adaptive infiltration [66,67].

### 6.2 Neutrophil NETosis and Immunothrombosis

Neutrophils contribute to plaque destabilization and thrombotic complications through the release of NETs [68]. This process, known as NETosis, promotes pathology through two distinct axes:

● Structural Damage: Histone-mediated cytotoxicity directly injures vascular endothelial cells and VSMCs, accelerating the expansion of the necrotic core [69].

● Inflammatory Amplification: NETs act as scaffolds that recruit monocytes and amplify local cytokine release [70].

Evidence Priority: While the role of NETs in plaque rupture is well-established, the existence of a circulating APC-like neutrophil subset represents an emerging area of research. These cells, characterized by upregulated MHC-II and costimulatory molecules, suggest that neutrophils may also contribute to the adaptive immune dialogue in high-risk patients [69].

### 6.3 Mast Cells: Effectors of Fibrous Cap Weakening

Mast cells (MCs) are primarily associated with the advanced stages of plaque vulnerability. Accumulating in the plaque shoulders and adventitia, activated MCs undergo degranulation in response to local stress or immunoglobulin E–high-affinity IgE receptor I (IgE-FcεRI) signaling [69,71]. This release of potent proteases—specifically tryptase and chymase—degrades the extracellular matrix and activates MMPs, directly weakening the fibrous cap. Clinical correlations confirm that MC infiltration density peaks at sites of plaque rupture, identifying MC degranulation as a terminal event in the transition from stable to vulnerable lesions [69,72].

### 6.4 Innate Lymphoid Cells (ILCs): Exploratory Regulators

ILCs represent a rapid-response system typically involved in mucosal homeostasis [73]. In atherosclerosis, ILC subsets (ILC1, ILC2, and ILC3) are thought to modulate the early inflammatory microenvironment through the secretion of IFN-γ, IL-5/IL-13, and IL-22, respectively [74].

Critical Insights: Despite single-cell multi-omics confirming their presence in human carotid plaques, direct causal evidence linking specific ILC subsets to human plaque progression remains preliminary [69,74]. Current hypotheses suggest they function as “sentinels” that influence early immune cell recruitment and endothelial function before the establishment of a robust adaptive response [3,4,75].

## 7. Integrated Molecular Networks: Orchestrating the Immune Dialogue

The progression of atherosclerosis is driven by a sophisticated molecular network where cytokines, metabolic intermediates, and epigenetic modifications converge to dictate immune cell fate and plaque stability [76].

### 7.1 Spatiotemporal Signaling: Cytokine and Chemokine Cascades

The immune landscape of the plaque is shaped by a hierarchy of soluble mediators. Early recruitment is governed by chemokines (e.g., CCL2, CX3CL1) that guide monocyte and T-cell infiltration via receptors such as CCR2 and CX3CR1 [14]. Once established, pro-inflammatory cytokines like TNF-α and IL-1β initiate a self-amplifying cascade, inducing endothelial activation and further leukocyte recruitment [77]. This network is further modulated by immune checkpoints (e.g., PD-1/PD-L1, TIGIT), which serve as critical rheostats; their dysfunction or therapeutic inhibition disrupts the balance of immune tolerance, leading to the rapid conversion of stable lesions into high-risk, vulnerable plaques [78,79,80].

### 7.2 Innate Sensing: PRRs and Inflammasome Activation

Pattern recognition receptors (PRRs) act as the primary sensors for “danger signals” within the necrotic core. Toll-like receptors (notably TLR4) recognize damage-associated molecular patterns (DAMPs) such as oxLDL and heat shock proteins, triggering the NF-κB and mitogen-activated protein kinase (MAPK) pathways to drive pro-inflammatory gene expression [81]. Crucially, the NLRP3 inflammasome integrates multiple stress signals, including cholesterol crystals and mitochondrial reactive oxygen species (ROS). Its activation leads to the proteolytic maturation of IL-1β and IL-18, directly promoting vascular wall injury and plaque instability [82]. The targeting of this TLR-NLRP3-IL-1β axis currently represents one of the most promising avenues for precision immunomodulation in ASCVD [83,84].

### 7.3 The Immuno-Metabolic Hub and Phenotypic Plasticity

Immune cell function is inextricably linked to cellular metabolism. In the oxygen-deprived and lipid-rich environment of the plaque, macrophages and effector T cells undergo metabolic reprogramming, shifting toward aerobic glycolysis to support rapid cytokine production [85]. This “metabolic switch” is not merely an energetic adaptation but a regulatory mechanism; metabolic intermediates themselves function as signaling molecules that reinforce the pro-inflammatory state [14]. Conversely, pathways such as liver X receptor/retinoid X receptor (LXR/RXR) signaling attempt to maintain homeostasis by coupling cholesterol efflux with the suppression of inflammatory genes [14]. The failure of this metabolic-immune axis is a fundamental driver of persistent chronic inflammation [17,86].

### 7.4 Epigenetic Memory and Trained Immunity

Recent evidence suggests that the “chronic” nature of vascular inflammation is sustained by epigenetic reprogramming, a phenomenon known as trained immunity [87]. Exposure to OSEs or high-glucose environments induces persistent epigenetic imprints—such as H3K4me3 and H3K27ac—at the promoters of key inflammatory genes (TNF, IL-6) [78]. These modifications enable innate immune cells to mount an exaggerated, hyper-inflammatory response upon subsequent stimulation [78]. This “inflammatory memory” is often transmitted to myeloid progenitors in the bone marrow, ensuring a continuous supply of pre-activated monocytes to the lesion [88]. Targeting the enzymes responsible for these modifications, such as histone methyltransferases or non-coding RNA regulators, offers a potential strategy for “resetting” the immune landscape of the vascular wall [89,90].

## 8. Precision Immunotherapy: From Bench to Vascular Clinic

Translating the functional heterogeneity of immune subsets into clinical outcomes requires a shift from broad immunosuppression to precision immunomodulation. While several strategies have shown promise in preclinical models, a significant “translational gap” remains due to the complex, spatiotemporal nature of human plaque evolution [18]. Representative targets, intervention strategies, research phases, and anticipated outcomes are summarized in Table 1.

**Table 1.** **Emerging precision immunotherapeutic targets and strategies for atherosclerosis**.

Target	Intervention method	Research phase	Key outcomes
PD-1/PD-L1 signaling pathway	Immune checkpoint inhibitors	Preclinical	Regulates macrophage polarization, inhibits M1 pro-inflammatory phenotype activation, promotes M2 anti-inflammatory phenotype conversion; reduces T cell exhaustion, decreases pro-inflammatory cytokine release, and alleviates vascular wall inflammation
Macrophage polarization-related proteins	Proteolysis-targeting chimeras (PROTAC)	Preclinical	Specifically degrades key proteins regulating macrophage polarization, achieves precise modulation of M1/M2 phenotypes
Metabolic reprogramming (e.g., lactate metabolism)/Epigenetic modification (e.g., m^6^A methylation)	Natural active substances	Preclinical	Bidirectionally regulates inflammatory response intensity via lactate-mediated histone lactylation; indirectly balances macrophage polarization
MicroRNA (miRNA)	Macrophage membrane-coated targeted nanocarriers	Preclinical	Specifically delivers miRNA to lesion macrophages, effectively promotes M2 anti-inflammatory polarization, and stabilizes atherosclerotic plaques
S1PR3	S1PR3-targeted therapy	Preclinical	Regulates T cell migration and localization, inhibits T cell exhaustion, reshapes the local immune microenvironment of plaques, and restores immune cell functional balance
CD19	CD19-targeted chimeric antigen receptor T (CAR-T) cell therapy	Preclinical	Specifically depletes pathogenic B cell clones, reduces autoimmune responses and pro-inflammatory antibody production
CD19 + PD-1/PD-L1 signaling pathway	CD19-targeted CAR-T therapy combined with immune checkpoint blockade	Preclinical	Depletes pathogenic B cell clones while reversing T cell exhaustion and restoring immune killing function
B cell activating factor (BAFF)	Biologics (e.g., Belimumab)	Clinical (approved for autoimmune diseases)	Blocks the binding of BAFF to B cell surface receptors, inhibits B cell survival, proliferation, and antibody production; provides cross-field reference for atherosclerosis treatment
B cell receptor (BCR) signaling pathway	Bruton’s tyrosine kinase (BTK) inhibitors	Preclinical	Selectively regulates the BCR signaling pathway, blocks downstream activation cascades, and inhibits excessive B cell activation and pathogenic antibody secretion
CD40-CD40L interaction	CD40-CD40L binding blockers	Preclinical	Inhibits T cell-dependent B cell activation, reduces the release of pro-inflammatory factors and autoantibodies, and alleviates the disorder of the plaque inflammatory microenvironment
Macrophage polarization regulators (e.g., miR-100-5p) + Anti-inflammatory factors (e.g., IL-10)	Macrophage membrane-coated nanoparticles (co-loading)	Preclinical	Synchronously induces macrophages to convert to M2 anti-inflammatory phenotype, promotes regulatory T cell (Treg) expansion, and dual-regulates the immune microenvironment
PD-1 + ALK (macrophage reprogramming-related molecules)	PD-1 inhibitors combined with macrophage reprogramming agents (e.g., ALK inhibitors)	Preclinical	Synchronously regulates macrophage polarization (inhibits M2 phenotype, promotes M1 phenotype) and recruits/activates CD8^+^ effector T cells, enhancing anti-tumor-like immune responses

IL-10, interleukin-10; m^6^A, N^6^-methyladenosine; PD-1, programmed cell death protein 1; PD-L1, programmed death-ligand 1; S1PR3, sphingosine-1-phosphate receptor 3; ALK, anaplastic lymphoma kinase.

### 8.1 Functional Reprogramming of the Myeloid Landscape

Rather than simply inhibiting macrophage infiltration, contemporary strategies aim to reprogram their functional state within the plaque.

● Epigenetic and Metabolic Modulation

Small molecules targeting histone lactylation or m^6^A methylation represent an emerging frontier for “resetting” the inflammatory memory of trained immunity. For instance, facilitating the transition toward a pro-resolving phenotype (formerly termed M2) can enhance efferocytosis and stabilize the necrotic core [80].

● Nanotechnology

Targeted nanocarriers, particularly those coated with leukocyte membranes, allow for the site-specific delivery of miRNAs or small molecules [91,92].

### 8.2 T-Cell Engineering and the Checkpoint Paradox

Restoring the balance between cytotoxic effectors and Tregs is a primary goal of precision cardiovascular therapy.

● Chimeric antigen receptor (CAR)-Treg Therapy: By engineering Tregs to specifically home to atherosclerotic lesions, researchers have successfully suppressed local inflammation without causing systemic immune deficiency [93,94].

● Modulating the Checkpoint Rheostat: PD-1/TIGIT signaling is a logical target. Future strategies must focus on checkpoint agonism or tissue-specific delivery to restore vascular tolerance without compromising anti-tumor immunity [95,96].

### 8.3 Selective B-Cell Modulation: From Depletion to Tolerance

B-cell-targeted therapies must distinguish between protective B1-derived IgM signals and pathogenic B2-derived IgG responses [49,51].

● Subset-Specific Depletion: While CD19-targeted CAR-T therapy can effectively deplete pathogenic B-cell clones, clinical application in ASCVD must be approached with caution to avoid losing the protective effects of Bregs [97,98].

● Targeting the B-T Axis: Biologics such as Belimumab (targeting B cell activating factor (BAFF)) or Bruton’s tyrosine kinase (BTK) inhibitors offer a means to dampen excessive B-cell activation and pathogenic antibody production [99]. Such approaches may be particularly relevant in post-myocardial infarction settings, where B2-cell-mediated inflammation accelerates secondary atherosclerosis [52].

### 8.4 The Future of Integrated, Multi-Target Synergies

The inherent redundancy of the immune system suggests that single-target therapies may be insufficient. The next generation of cardiovascular immunotherapy likely lies in multi-modal synergy [100,101]:

● Spatiotemporal Delivery: Utilizing nanoparticles to co-load pro-resolving agents (e.g., IL-10) with metabolic regulators, simultaneously inducing macrophage reprogramming and Treg expansion [102,103].

● Combinatorial Checkpoint/Metabolic Blockade: Coupling the reversal of T-cell exhaustion with the inhibition of glycolytic pathways to shift the entire plaque microenvironment from a “combustive” to a “resolving” state [104,105].

## 9. Challenges and Future Directions: From Multi-Omics Discovery to Clinical Precision

While single-cell technologies have revolutionized our understanding of vascular immunology, translating these high-resolution “cellular catalogs” into clinical benefit remains a formidable challenge. Future research must evolve from descriptive mapping toward a functional, integrated understanding of immune plasticity [106].

### 9.1 Balancing Interspecies Differences and Vascular Bed Heterogeneity

A significant limitation of current research is the over-reliance on murine models, which exhibit fundamental immunological deviations from human atherosclerosis [107]:

● Cellular Composition: Murine plaques are predominantly macrophage-driven, whereas human lesions are characterized by a higher density of T-cell lineages. This discrepancy undermines the reliability of mice in modeling fibrous cap thinning and plaque rupture—the terminal events of human disease [108].

● Anatomical Heterogeneity: Immune signatures are not interchangeable across vascular beds. Hemodynamic forces and local endothelial phenotypes dictate specific immune responses in the coronary versus carotid arteries. Future precision therapies must be tailored to both the disease stage (e.g., fatty streak vs. necrotic core) and the distinct anatomical territory [109].

### 9.2 Integrated Multi-Omics: Beyond Transcriptomic “Snapshots”

The identification of immune subsets via scRNA-seq represents only a “discovery phase”. To achieve clinical relevance, these data must be integrated into a broader functional framework [110]:

● Evidence Prioritization: There is an urgent need to bridge the gap between transcriptomic observations and spatial reality. Utilizing advanced algorithms like SSGATE to couple epigenomic and proteomic data with Spatial Transcriptomics will allow us to localize pathogenic subsets (e.g., SPP1^+^ macrophages) within specific plaque niches [111].

● Establishing a Reference Atlas: Standardizing data across global cohorts is essential for identifying robust, reproducible targets that transcend individual study noise [112].

### 9.3 Biomarkers for Personalized Immunotherapy

The clinical translation of immune subsets requires non-invasive, high-specificity biomarkers:

● Dynamic Monitoring: Integrating T-cell receptor (TCR) clonotypes and metabolic imprints will enable the development of “liquid biopsy” panels. These tools are critical for predicting treatment responses and monitoring the “Athero-Oncology” risk in patients undergoing ICI therapy [113,114].

### 9.4 Spatiotemporally Specific Delivery Systems

To overcome the limitations of systemic immunosuppression, drug delivery must become “environmentally aware” [115]:

● Stimuli-Responsive Nanocarriers: Future systems should leverage the unique plaque microenvironment—sensing pH shifts or MMP activity—to trigger localized drug release [116].

● Stage-Specific Intervention: Immunomodulation should follow the disease’s natural history: inhibiting recruitment during lesion initiation and promoting efferocytosis and matrix repair during the vulnerable stage [117,118].

## 10. Barriers to Clinical Translation: Bridging the “Translational Divide”

The slow pace of clinical translation in cardiovascular immunology, compared to oncology, stems from several unique barriers:

● Phenotypic Plasticity: The high plasticity of immune cells means that an intervention intended to suppress a pathogenic subset might inadvertently impair a closely related, protective subset [119,120].

● Imaging Limitations: We currently lack clinical imaging modalities capable of providing real-time, *in vivo* molecular signatures of immune cell function within the vessel wall [121,122].

**Fig. 1.**
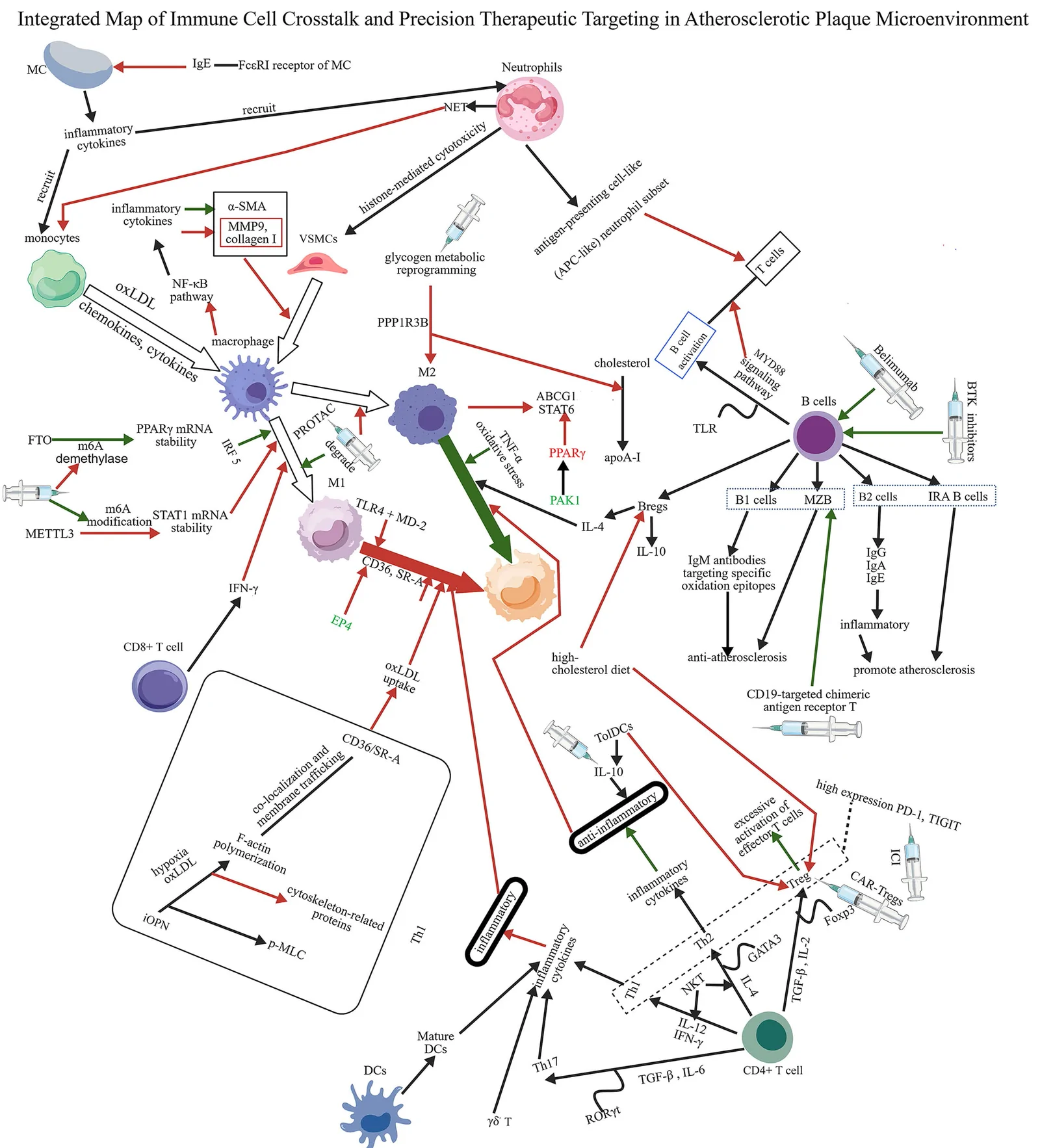
**Integrated map of immune cell crosstalk and precision therapeutic targets in atherosclerosis**. ABCA1, ATP-binding cassette transporter A1; ABCG1, ATP-binding cassette transporter G1; APC, antigen-presenting cell; apoA-I, apolipoprotein A-I; α-SMA, alpha-smooth muscle actin; Bregs, regulatory B cells; BTK, Bruton’s tyrosine kinase; CAR-Tregs, chimeric antigen receptor regulatory T cells; CD4, cluster of differentiation 4; CD8, cluster of differentiation 8; CD19, cluster of differentiation 19; CD36, cluster of differentiation 36; DCs, dendritic cells; EP4, prostaglandin E receptor 4; F-actin, filamentous actin; FcεRI, high-affinity immunoglobulin E receptor; Foxp3, forkhead box P3; FTO, fat mass and obesity-associated protein; GATA3, GATA-binding protein 3; ICI, immune checkpoint inhibitor; IFN-γ, interferon gamma; IgA, immunoglobulin A; IgE, immunoglobulin E; IgG, immunoglobulin G; IgM, immunoglobulin M; IL-2, interleukin-2; IL-4, interleukin-4; IL-6, interleukin-6; IL-10, interleukin-10; IL-12, interleukin-12; iOPN, intracellular osteopontin; IRA B cells, innate response activator B cells; IRF5, interferon regulatory factor 5; M1, classically activated macrophage; M2, alternatively activated macrophage; m^6^A, N^6^-methyladenosine; MC, mast cell; MD-2, myeloid differentiation factor 2; METTL3, methyltransferase-like 3; MMP9, matrix metalloproteinase 9; mRNA, messenger RNA; MyD88, myeloid differentiation primary response 88; MZB, marginal zone B cell; NET, neutrophil extracellular trap; NF-κB, nuclear factor kappa B; NKT, natural killer T cell; oxLDL, oxidized low-density lipoprotein; PAK1, p21-activated kinase 1; PD-1, programmed cell death protein 1; p-MLC, phosphorylated myosin light chain; PPARγ, peroxisome proliferator-activated receptor gamma; PPP1R3B, protein phosphatase 1 regulatory subunit 3B; PROTAC, proteolysis-targeting chimera; RORγt, retinoic acid receptor-related orphan receptor gamma t; SR-A, scavenger receptor class A; STAT1, signal transducer and activator of transcription 1; STAT6, signal transducer and activator of transcription 6; TGF-β, transforming growth factor beta; Th1, T helper 1 cell; Th2, T helper 2 cell; Th17, T helper 17 cell; TIGIT, T-cell immunoreceptor with immunoglobulin and ITIM domains; TLR, Toll-like receptor; TLR4, Toll-like receptor 4; TNF-α, tumor necrosis factor alpha; TolDCs, tolerogenic dendritic cells; Tregs, regulatory T cells; VSMCs, vascular smooth muscle cells; γδ T, gamma delta T cell. Created with biogdp (https://biogdp.com/zh). Red arrows indicate activating/pro-inflammatory or pro-atherogenic effects; green arrows indicate inhibitory/anti-inflammatory or atheroprotective effects; black arrows indicate general biological interactions or signaling relationships without assignment of an overall pro- or anti-atherogenic effect. Solid arrows denote principal or established relationships, whereas dashed arrows denote indirect or proposed regulatory interactions. Arrow thickness is used for visual emphasis only and does not indicate quantitative effect size or strength of evidence.

● The Safety-Efficacy Trade-off: Long-term modulation of pathways like IL-1β or NLRP3 must be balanced against the risk of impaired host defense. Achieving “vascular-specific” tolerance without systemic compromise remains the ultimate goal of precision cardiovascular medicine [123,124,125]. The integrated immune-cell crosstalk and corresponding precision therapeutic targets are summarized in Fig. 1.

## 11. Conclusions

The paradigm of atherosclerosis has fundamentally shifted from a passive lipid-storage disorder to a highly dynamic, immune-mediated process. As established throughout this review, the functional heterogeneity of immune cell subpopulations—ranging from the phenotypic plasticity of macrophages to the intricate regulatory axes of T and B cells—dictates the transition from stable lesions to clinical cardiovascular events. By moving beyond static cellular classifications and embracing a stage-specific understanding of the immune microenvironment, we can better identify the molecular “tipping points” that lead to necrotic core expansion and fibrous cap destabilization.

However, the path toward precision cardiovascular immunotherapy is marked by several biological paradoxes, most notably the “Athero-Oncology” challenge. The clinical observation that immune checkpoint inhibition for cancer can inadvertently accelerate plaque progression underscores the necessity for site-specific rather than systemic immunomodulation. Emerging technologies, such as single-cell multi-omics and chimeric antigen receptor regulatory T-cell (CAR-Treg) engineering, provide the tools necessary to bridge the translational gap between murine models and human pathology.

Ultimately, the future of atherosclerosis management lies in the ability to “reset” the vascular immune landscape. This involves targeting trained immunity and metabolic reprogramming to restore homeostatic balance within the vessel wall. While substantial barriers to clinical translation remain—particularly regarding the real-time monitoring of plaque inflammation—the shift toward subset-specific precision therapy offers a promising frontier for reducing the global burden of atherosclerotic cardiovascular disease.
